# Angiogenesis and immune checkpoint dual blockade in combination with radiotherapy for treatment of solid cancers: opportunities and challenges

**DOI:** 10.1038/s41389-021-00335-w

**Published:** 2021-07-10

**Authors:** Lingling Zhu, Xianzhe Yu, Li Wang, Jiewei Liu, Zihan Qu, Honge Zhang, Lu Li, Jiang Chen, Qinghua Zhou

**Affiliations:** 1 Lung Cancer Center, West China Hospital of Sichuan University, Chengdu, 610041 Sichuan Province China; 2 Department of Radiation Oncology, Massachusetts General Hospital, Harvard Medical School, Boston, 02114 MA USA; 3 Gastrointestinal Department, The Affiliated Hospital of Zunyi Medical University, Zunyi, Guizhou Province People’s Republic of China; 4 Department of General Surgery, Sir Run Run Shaw Hospital, Zhejiang University, Hangzhou, 310016 Zhejiang Province China

**Keywords:** Radiotherapy, Cancer immunotherapy

## Abstract

Several immune checkpoint blockades (ICBs) capable of overcoming the immunosuppressive roles of the tumor immune microenvironment have been approved by the US Food and Drug Administration as front-line treatments of various tumor types. However, due to the considerable heterogeneity of solid tumor cells, inhibiting one target will only influence a portion of the tumor cells. One way to enhance the tumor-killing efficiency is to develop a multiagent therapeutic strategy targeting different aspects of tumor biology and the microenvironment to provide the maximal clinical benefit for patients with late-stage disease. One such strategy is the administration of anti-PD1, an ICB, in combination with the humanized monoclonal antibody bevacizumab, an anti-angiogenic therapy, to patients with recurrent/metastatic malignancies, including hepatocellular carcinoma, metastatic renal cell carcinoma, non-small cell lung cancer, and uterine cancer. Radiotherapy (RT), a critical component of solid cancer management, has the capacity to prime the immune system for an adaptive antitumor response. Here, we present an overview of the most recent published data in preclinical and clinical studies elucidating that RT could further potentiate the antitumor effects of immune checkpoint and angiogenesis dual blockade. In addition, we explore opportunities of triple combinational treatment, as well as discuss the challenges of validating biomarkers and the management of associated toxicity.

## Introduction

Although immune checkpoint blockades (ICBs) that target programmed cell death 1 receptor (PD-1)/programmed cell death receptor ligand 1 (PD-L1), and cytotoxic T lymphocyte antigen 4 (CTLA-4) axis have rapidly transformed the anticancer therapy and drug development landscape [1], the general response rate remains unsatisfactory [2]. Evidence suggests that anti-angiogenesis agents function as ideal partners for ICBs [3]. In fact, combination anti-angiogenic therapy and ICBs were approved by the US Food Drug Administration (FDA) for the treatment of multiple solid cancers [4], including hepatocellular carcinoma (HCC) [5], metastatic renal cell cancer [6], non-small cell lung cancer (NSCLC) [7], metastatic endometrial cancer [8], and uterine cancer (Table 1). For instance, a successful phase III trial (the IMbrave150 trial) for unresectable HCC reported a progression-free survival (PFS) of 6.8 months vs. 4.3 months, as well as an objective response rate (ORR) of 27% vs. 12%, in the atezolizumab plus bevacizumab and sorafenib arms, respectively [9]. The results of this clinical trial contributed to the approval of atezolizumab plus bevacizumab as a first-line therapy for unresectable HCC. Despite this breakthrough resulting in significantly improved patient outcomes for certain types of cancer, nearly two-thirds of patients remain unresponsive, likely owing to low immunogenicity [3,10,].

**Table 1:** US Food Drug Administration-approved immune checkpoint blockade plus anti-angiogenic therapy in solid cancer.

Disease setting	Agents	Clinical trials gov number/trial name (if applicable)	FDA approval	Primary outcome	Study Phase	Approved year	Most common grade ≥ 3 AEs	References
HCC	Atezolizumab + bevacizumab vs	NCT02715531	Advanced HCC	Partial responses 62%	I	2020	Hypertension 15.2%	[5]
MRCC	Avelumab + axitinib vs Sunitinib	NCT02684006/ JAVELIN Renal 101	Advanced clear cell RCC	Median PFS (16.6 vs 11.2mo) in patients irrespective of PD-L1 expression, MedianOS (not reached)	III	2019	Hand-foot syn drome (9% vs. 9%), hypertension (30% vs. 18%), platelet count decreased (0% vs. 32%)	[6]
NSCLC	Atezolizumab + chemotherapy + bevacizumab vs chemotherapy + bevacizumab	NCT02366143/Impower 150	First-line treatment of stage IV non-squamous NSCLC	Median OS (19.2 vs 14.7; HR: 0.78; 95% CI: 0.64, 0.96; *p* = 0.016)	III	2018	Grade 3–4 AEs (57% vs 49%); grade 5 AEs (3% vs 2%)	[7]
Metastatic endometrial carcinoma	Pembrolizumab + lenvatinib	NCT02501096/ KEYNOTE 146	Metastatic endometrial carcinoma	ORR at week 24: 39·6% (95% CI 26·5–54·0)	I/II	2018	Hypertension (34%); diarrhea (8%)	[8]
Uterine cancer	Cabozantinib + atezolizumab	NCT03170960/ COSMIC-021	Inoperable, locally advanced, metastatic, or recurrent uterine cancer	ORR: 27%,	Ib	2020	57%, no grade 5 AEs	–

*HCC* hepatocellular carcinoma, *mRCC* metastatic renal cell carcinoma, *NSCLC* non-small cell lung cancer, *FDA* Food and Drug Administration, *PFS* progression-free survival, *ORR* objective response rate, OS overall survival, CI confidence interval, AEs adverse events.

Radiotherapy (RT) is applied in more than 60% of cases with malignancies throughout the course of treatment [11]. Therefore, the addition of another treatment approach, particularly RT, could further augment the antitumor efficacy of the dual combination therapeutic strategy of anti-angiogenesis plus ICBs [10], in part due to the interplay and synergies between the tumor vasculature and antitumor immunity within the tumor immune microenvironment (TME) [10]. On the one hand, RT can reprogram a “cold” TME to an immune-reactive, “hot” one [12]. On the other hand, angiogenic agents can normalize tumor vessels and potentiate the efficiency of RT by forming an immunology-favoring tumor microenvironment. Moreover, PD-1/PD-L1 inhibitors can reverse RT-mediated exhaustion pathways, while anti-CTLA-4 therapy relieves the inhibitory signals from antigen-presenting cells (APCs) and regulatory T cells (Tregs) induced by RT [13]. In addition, in a Lewis lung mouse model, indoleamine 2,3‑dioxygenase (IDO) inhibitor plus RT therapy synergistically reduced the proportion of Tregs and downregulated the levels of exhaustion molecules, including PD-1, PD-L1, and T cell immunoglobulin domain and mucin domain 3 (TIM3), on dendritic cells (DCs) and T cells [14]. Hence, ICBs can overcome the upregulated inhibitory molecules and pathways triggered by RT and restore T cell activity, which enhances the synergistic efficiency of RT [13]. Although a multimodal approach for cancer management is no longer a novel concept, the combinational therapy of ICBs, RT, and anti-angiogenesis remains a therapeutic innovation yet to achieve promising clinical benefits.

The clinical outcomes of the dual combination vary across malignancies, with an ORR of 30.8% for advanced HCC, 36% for colorectal cancer (CRC), 39.6% for advanced endometrial cancer, 44% for gastric cancer, 48.3% for mucosal melanoma, 72.7% for advanced NSCLC, and 73% for mCRC [3]. These variable effects may result from different tumor types with varied immunogenicity, leading to varying responses to diverse antigens [3] and distinct intra-tumor heterogeneous cells with variable molecular, genetic, and phenotypic properties [15]. For example, pancreatic ductal adenocarcinoma is characterized by a rich heterogeneous desmoplastic stroma, including immune cells, stromal fibroblasts, endothelial cells, neurons, and collagen deposition [16]. Moreover, the tumor heterogeneity associated with CRC serves to modulate cancer progression and metastasis and is involved in the cellular hierarchy, clonal diversity, and establishment of the TME, including determining the location and function of immune cells [17]. In terms of immune infiltration, a heterogeneous mixture of immune cells consists of both innate and adaptive immune cell subsets, linked to active and suppressive functions [18]. In addition, a heterogeneous tumor microenvironment might correlate with a disordered blood vessel network, probably resulting in variable niches, such as hypoxic or perivascular regions [19]. As a result, heterogeneity may lead to an uneven distribution of genetically diverse cell subpopulations and variable vasculature across and within disease sites [15].

However, despite the increasing number of cases being treated with combination RT, anti-angiogenic therapy, and ICBs, on the basis of tumor type and TME, certain limitations and challenges exist that must be addressed. Here, we aim to summarize and compare recent preclinical and clinical results of RT/ICBs/antiangiogenic therapies across diverse cancer types, with particular attention paid to mechanistic rationale. We also review evidence that explores the optimal choice, sequencing, and timing of RT/ICBs/antiangiogenic therapies, acquired resistance, biomarkers for patient selection, and potential toxicity.

## Rationale for dual blockades of immune checkpoint and angiogenesis

**Fig. 1:**
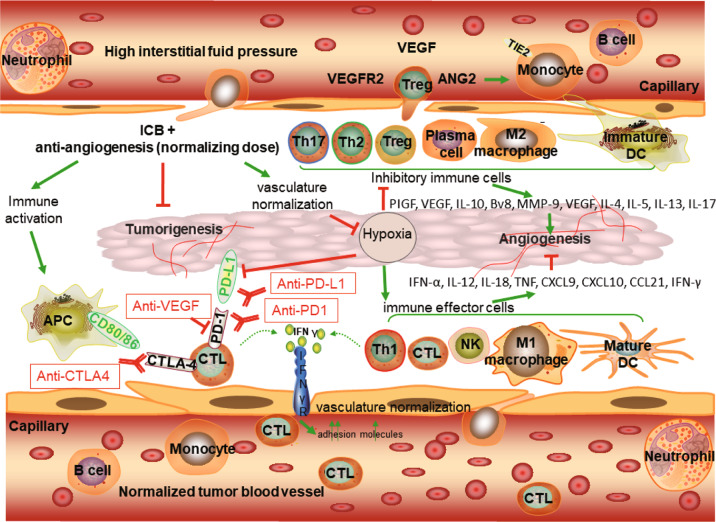
Mechanistic rationale for immune checkpoint blockade in combination with anti-angiogenic agents. Combinatorial therapy activates the immune response and suppresses the inhibitory immune signals by decreasing the expression of multiple immune checkpoints, increasing the ratio of anti-/pro-tumor immune cells, and alleviating hypoxia by normalizing tumor vasculature.

Angiogenesis factors, including vascular endothelial growth factor (VEGF) and angiopoietin 2 (ANG2), contribute to immune suppression via repressing APCs and other antitumor immune effector cells, or via potentiating the function of immunosuppressive Tregs, myeloid-derived suppressor cells (MDSCs), and M2-tumor-associated macrophages (TAMs) [2]. These immunosuppressive cells can subsequently stimulate angiogenesis, thereby creating a cycle conducive to impaired immune activity [20]. A judicious dose of anti-angiogenic agents not only prunes blood vessels that are pivotal for tumor progression, but also blocks negative immune signals by decreasing the level of immune checkpoints, thereby increasing the anti-/pro-tumor immune subset ratio and alleviating hypoxia by normalizing tumor vasculature. Therefore, appropriate anti-angiogenesis administration can alleviate immunosuppression and enhance immunity, thereby improving the efficacy of ICBs [2]. Moreover, ICBs might increase the efficacy of anti-angiogenic therapies by recruiting immune cell subtypes with angio-modulatory function [21], providing a strong rationale for developing the dual combination. For example, T helper 17 (TH17) subset of TH cells promotes angiogenesis by secreting placental growth factor (PlGF) [22] (Fig. 1).

Vascular abnormalities of solid tumors lead to a TME characterized by hypoxia, low extracellular pH, and increased interstitial fluid pressure [2]. Hypoxia compromises the functionality of immune effector cell types, such as natural killer T cells, M1-type TAMs, mature DCs, and TH1 cells, and promotes immunosuppressive immune cell recruitment, such as Tregs, MDSCs, and M2-type TAMs [23,24,]. Reduced hypoxia, via judicious dosing of anti-angiogenesis agents (normalizing dose), induces vessel normalization, which has been reported to promote macrophage polarization to the immune-supportive M1 phenotype and enhance infiltration of CD8+ cytotoxic T cells into tumors in HCC [25]. Alleviated hypoxia can also promote other immunostimulatory phenotypes and reduce the immunosuppressive phenotypes of immune cells, thereby influencing angiogenesis via different cytokines or chemokines, including interferon (IFN)-α, interleukin (IL)−12, IL-18, and tumor necrosis factor-α from mature DCs; CXCL9, CXCL10, and CCL21 from M1-like TAMs; IFN-γ from CD8+ T and TH1 cells; VEGF, IL-10, Bv8, and matrix metallopeptidase (MMP)−9 from immature DCs, MDSCs, M2 TAMs, and TIE2-expressing macrophages; and VEGF, IL-4, IL-5, IL-13, and IL-17 from Tregs, TH2, and TH17 cells [4]. Reduced hypoxia also downregulates the level of PD-1 on CTLs and PD-L1 on tumor cells, while blocking VEGF signaling [21]. Furthermore, decreased hypoxia via administration of anti-angiogenic drugs can effectively inhibit the suppressive signal for DC maturation, reduce the recruitment of Tregs, and reduce the number of MDSCs, as well as their effectiveness [26]. In addition, the vascular endothelial growth factor receptor-2 (VEGFR2) inhibitor apatinib reportedly contributes to anti-PD-1 efficiency in mice with colon cancer by enhancing PD-L1 level [27]. Our previous research also demonstrated that anti-VEGFR2 augmented PD-1 levels on CD4+ cells and the activity of cytotoxic CD8+ T cells and contributed to a reduction in infiltrating Tregs and monocytes [28].

Notably, the effect of anti-angiogenic therapies on the TME varied among different anti-angiogenic compounds, including the humanized monoclonal antibody targeting VEGF bevacizumab, the tyrosine kinase inhibitors sunitinib, sorafenib, imatinib, dasatinib, nilotinib, and the proteasome inhibitor bortezomib [29]. For instance, sunitinib appears to be an acceptable option because it can augment the function of APCs and T cells through reducing the number of Tregs and MDSCs [29]. However, Alfaro et al. reported that bevacizumab and sorafenib, rather than sunitinib, seemed to improve the function of APCs such as DCs [30]. In a melanoma model, regorafenib has the most potent function, among a number of kinase inhibitors, in enhancing antitumor immunity via inhibiting IFN-γ-induced expression of PD-L1 and Indoleamine 2,3-dioxygenase 1 through RET–Src inhibition [31].

Moreover, the effects of VEGF antibodies, such as sorafenib, might be dose-dependent, with low dosages (normalizing dose) tending to trigger vessel normalization, diminish hypoxia, and enhance antitumor immunity (beneficial effects), whereas a high dose might tend to potentiate hypoxia and enhance immunosuppression (detrimental effects) [32]. Activated angiopoietin 2 (ANG2) signaling reportedly contributes to immunosuppression by increasing leukocyte–endothelial interplay by stimulating adhesion molecules, ultimately promoting the recruitment of MDSCs, Tregs, and TIE2-expressing monocytes in vitro [2]. In addition, ipilimumab plus bevacizumab has been demonstrated to be correlated to downregulated tumor levels of ANG2, which may be the reason for the inhibitory effect of bevacizumab on the VEGF effect in enhancing ANG2 expression in tumors [33]. The dual combinatorial approach also impacts the immune memory response. For instance, a phase I clinical trial of combined bevacizumab and ipilimumab revealed a ≥50% increase in the number of circulating CD4+ and CD8+ memory cells in patients with unresectable stage III or IV melanoma [34]. Therefore, targeting VEGF, VEGR2, or ANG2 may enhance vessel normalization, while facilitating anticancer immunity, thus exerting synergistic effects when combined with anti-PD-1 [35].

ICB therapy may also restore the immune-supportive microenvironment by inhibiting immune checkpoints and promoting vessel normalization in a T cell-dependent manner [2] (Fig. 1). For example, TH1 cells, upon activation by ICB, secrete interferon-γ (IFN-γ) and then directly potentiate intercellular adhesion molecule (ICAM)−1 and drive T cell migration, thereby promoting tumor vascular normalization and regression via the IFN-γ receptor on cancer endothelial cells [36], ultimately inhibiting tumor growth, enhancing vessel perfusion, and decreasing intratumoral hypoxia [37]. In addition, ipilimumab (anti-CTLA-4) in conjunction with bevacizumab (anti-VEGF) therapy significantly upregulated adhesion molecules, such as CD31, E-selectin, and VCAM-1, on intratumoral endothelial cells in metastatic melanoma patients, while promoting adhesion of activated T cells to tumor-associated endothelial cells [38]. In addition, hypoxia-driven inhibitory immune signals, such as PD-L1 on macrophages, DCs, and tumor cells, can become blocked by PD-L1 inhibitors [39].

Overall, anti-angiogenesis therapy and ICB combination approaches develop a positive reinforcing feedback loop to normalize tumor blood vessels, relieve hypoxia via increased tumor perfusion and enhance the activation and infiltration of effector T cells, thus providing survival benefits [40]. However, the rates and durability of the response to combination therapy require further improvement. Specifically, there is a need for the addition of another treatment modality to increase the effectiveness of the dual combination strategy and further improve patient outcomes.

## Mechanistic rationale for adding RT to dual combination immune checkpoint blockades and anti-angiogenic therapy

RT, a mainstay of first-line therapy for multiple solid tumors [12], exerts a direct effect on the tumor stroma, such as blood vessels and immune cells [41]. Combinations of radio-, immune-, or anti-angiogenic treatments have shown potential clinical benefits [10]. Here, we focus on the role of RT in normalizing tumor vasculature and augmenting the immune response when combined with ICB and anti-angiogenic agents according to the most recent data.

**Fig. 2:**
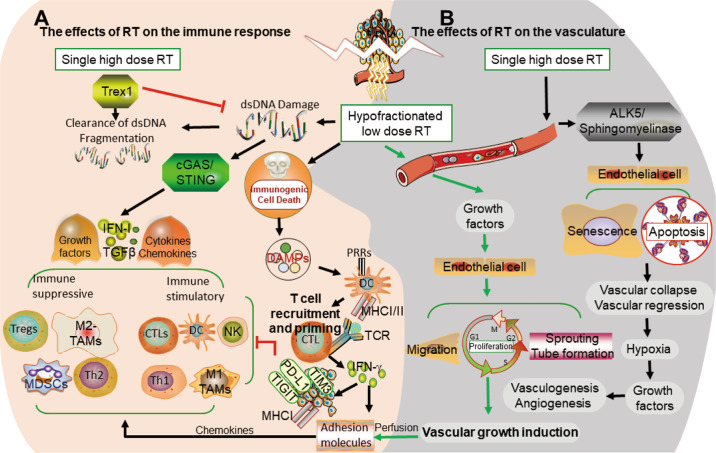
Potential role of RT (fractionated low dose versus single high dose) on the tumor vasculature, tumor cell, and microenvironment. **A** Main effects of RT on the immune response. High-dose RT triggers TREX1 resulting in clearance of cytosolic dsDNA. Multiple chemokines, cytokines, and growth factors secreted, upon RT, via cytosolic dsDNA/cGAS/STING signaling, promote the recruitment of immune cells. RT facilitates an immune response by inducing immunogenic cancer cell death and DAMPs, which activate antigen-presenting cells such as DCs PRRs, and prime CTLs, ultimately causing the release of cytokines, which not only exerts an immunosuppressive role by potentiating PD-L1 level on tumor cells but also drives immune cell recruitment by upregulating leukocyte adhesion molecules in the vessel wall. **B** Main effects of RT on the vasculature. Single high-dose RT triggers apoptosis and senescence of endothelial cells by upregulating ALK5 and sphingomyelinase, leading to vascular regression and collapse and eventual vasculogenesis and angiogenesis. Fractionated low-dose irradiation upregulates angiostimulatory growth factors, inducing vascular growth and tissue perfusion by potentiating diverse endothelial cell functions, such as migration, proliferation, and sprouting tube formation.

The observation of an abscopal effect, i.e., local radiation-mediated systemic tumor rejection, provided evidence of an interaction between the immune response and RT [42]. RT can potentiate antitumor immune responses via several mechanisms. One example is that DNA damage-induced cancer cell death promotes the expression of neoantigens and damage-associated molecular patterns (DAMPs), thereby promoting antigen presentation activity and specific T cell priming; another is that cytosolic double-stranded DNA (dsDNA) damage induced by RT can induce the release of multiple chemokines, cytokines, and growth factors via dsDNA/cyclic GMP–AMP synthase (cGAS)/stimulator of interferon genes (STING) signaling, resulting in recruitment of immunosuppressive and immunostimulatory cells [12] (Fig. 2A).

The immunosuppressive effects of RT include recruitment of specific immune subsets and polarization of immune subsets into a pro-tumor phenotype, such as Tregs, MDSCs, TH2 cells, TH2-skewed CD4+T cells, and M2-TAMs [13]. In addition, RT can upregulate the level of immune checkpoint molecules, including PD-L1 on tumor cells [10], TIM3 on CD4+ [43], CD8+ T cells, and Tregs [44], as well as T cell immunoreceptor with Ig and ITIM domains (TIGIT) on CD8+ T cells, natural killer (NK) cells, Tregs, and T follicular helper cells [45], which effectively dampens antitumor activity. Other aspects of the immunosuppressive role of RT include triggering immunosuppressive chemokines and SDF1-α, CCL22, CCL28, CCL2 [46], TGFβ [47], and hypoxia-inducible factor 1-α (HIF-1α) [48]. In fact, in an orthotopic murine head and neck squamous cell cancer model, multiple layers of immune regulation, including concurrent blockade of TIM-3 and PD-L1 blockade with RT, or targeted Treg depletion, effectively overcame tumor resistance [44]. Moreover, in a mouse model of head and neck tumor cells, modulating the immunosuppressive TME was found to enhance the effectiveness of ICIs plus RT [49].

DNA damage-induced cancer cell death is a well-established mechanism responsible for the anticancer effects of RT. Immunogenic cell death renders dying tumor cells susceptible to the immune cell-mediated killing by releasing DAMPs [50], which then interact with pattern recognition receptors on APCs, including DCs, and then activate DC cross-present antigens and migrate to draining lymph nodes [51]. Therefore, theoretically, radiation fields including the tumor-draining lymph nodes might be detrimental for an antitumor immune response. However, Spratt et al. recommend the extension of the RT field to include the common iliac lymph nodes for patients deemed to need whole pelvic RT, among men with prostate cancer [52]. For breast cancer patients with a positive sentinel lymph node who elect to forgo an axillary dissection, and patients with drainage to the internal mammary lymph nodes, radiation treatment fields must be specifically designed to include the appropriate nodal regions within the target treatment volumes [53]. Two patients with alveolar extremity rhabdomyosarcoma had failed RT treatment because of untreated in-transit regional nodes [54]. Overall, designing radiation fields including draining of lymph nodes provides more comprehensive coverage of the regional lymph nodes at risk, but it might also increase the risk of dampening the immune cells in the lymph nodes. Thus, it is reasonable to individualize the planning target volume according to the circumstances to improve local control.

DsDNA delivered by CD11c-mediated endocytosis reportedly promotes DC maturation in vitro, depending on the cytoplasmic DNA-sensing cGAS/STING pathway [51]. The cGAS/STING pathway elicited the release of proinflammatory cytokines, chemokines, and growth factors [55], including type I IFN [56], upon RT-induced cytosolic dsDNA damage. Certain proinflammatory factors, such as CXCL9, CXCL10, and CXCL16, promote the trafficking of immune cells into the tumor, increase leukocyte chemoattraction and extravasation, and induce tumor surface expression of PD-L1, MHC-I, NK cell ligand, and Fas (CD95) [10,41,57,]. Upregulation of the Fas [58] and MHC-I level [47] in malignant cells following RT enhances T cell-mediated recognition of cancer cells. Similarly, upregulation of NKG2D ligand expression upon RT in cancer cells makes them susceptible to NK cell killing [59]. RT also activates NK cell-mediated tumor cell clearance through elevating the level of NKG2D receptor stress ligands [59]. Moreover, RT can augment antitumor immunity by triggering the upregulation of adhesion molecules on endothelial cells and chemokines secreted by cancer cells, both of which promote the extravasation of the immune cells into the tumor sites [60] (Fig. 2A).

However, the immunoregulatory response to RT is seemingly dependent on fractionation, dose, and timing [61]. Given that the biological effective dose (BED) is an in vitro concept that does not consider the effect of the TME, a practical concept of the immunologically effective dose (IED) for immuno-RT was proposed [61]. A hypofractionated dose (a threshold dose ≤8–10 Gy), instead of a high single-dose (20 Gy) radiation, is proposed to induce effective antitumor immunity, including abscopal responses and IFNβ activation, in different cancer cells [62]. Furthermore, despite having a similar BED, the IED efficacy of 3 × 8 Gy was more than twice that of the 5 × 6 Gy regimen in breast cancer [63], while a clinical trial (NCT02221739) showed that administration of either 5 × 6 Gy or 3 × 9 Gy with ipilimumab increased treatment response, with no significant difference observed in chemotherapy-refractory metastatic NSCLC cases [64].

It was also reported that a DNA exonuclease, three prime repair exonuclease 1 (TREX1), triggered by radiation doses above 12–18 Gy, could attenuate immunogenicity by degrading accumulated DNA in the cytosol following RT, leading to reduced abscopal effect [62]. For instance, a single dose administration of 20–30 Gy rather than a fractionated regimen (3 × 8 Gy) negatively impacted tumor immunogenicity and the abscopal effect in TSA mammary carcinoma, a mouse mammary carcinoma refractory to ICBs, and colorectal MCA38 mouse carcinoma models [62]. Moreover, fractionated (3 × 8 Gy), but not single-dose (1 × 20 Gy), RT plus CTLA-4 blockade established an abscopal response in mouse models of breast cancer and colon cancer [65]. In addition, in mice bearing CT26 colon or B16-F10 melanoma cancers, three different fractionated regimens with similar BED (1 × 16.4, 3 × 8, and 18 × 2 Gy) were investigated [45]. The 3 × 8 Gy scheme was the most effective when administered with blockades of both TIGIT and PD-L1, due to increased levels of PD-L1 and TIGI, while the 18 × 2 Gy scheme was effective with anti-PD-L1 because of the sustained upregulation of PD-L1 levels. Similarly, in cases with untreated melanoma brain metastases, a fractionation regimen of 3 × 9 Gy showed more favorable intracranial PFS compared to a single dose of 18–20 Gy (70% vs. 46% at 6 months; *P* = 0.01), especially when combined with nivolumab [66]. However, in a preclinical study of melanoma, a single fraction of 15 Gy enhanced tumor immune cell infiltration compared to a fractionated (3 × 5 Gy) schedule [67]. Similarly, in in vivo and in vitro models of triple-negative breast cancer, the primary steps of RT-derived antitumor immune priming are preferentially stimulated via a single dose of 20 Gy rather than radiation regimens (4 × 2 Gy, 2 Gy, 0 Gy) [60]. This may be caused by shorter delivery periods, which impede continued eradication of tumor-infiltrating immune cells in some immunologically sensitive tumors, such as murine CT26 and MC38 colon tumors [68]. A mathematical model simulation further revealed that the optimal RT dose per fraction for a maximal antitumor response was 10–13 Gy [69]. For instance, PD-L1 blockade plus 12 Gy RT achieved abscopal effects and superior local tumor inhibition of irradiated tumors in a mammary tumor mouse-derived xenograft model compared with IR or anti-PD-L1 monotherapy [70]. However, hypofractionated RT schedules, with extended periods during which treatment-stimulated T cells infiltrate the irradiated tumor, have been demonstrated to promote systemic antitumor effectiveness similar, but not inferior, to those with treatments with shorter schedules when combined with anti-PD1 in B16 melanoma and 4T1 breast carcinoma mouse models [71]. Despite these inconsistencies, all evidence indicates that responding immune cells are critical for an antitumor response during RT plus ICB combinatorial treatment. The inconsistencies may also result from the overall modulatory effect of RT being tissue- or tumor microenvironment-specific [10]. For example, the bone marrow is an immune-privileged site, a phenomenon in which certain sites are more likely to prevent antitumor immune attack after RT [72]. A phase I clinical study investigated the safety and efficacy of stereotactic body radiotherapy (SBRT) plus ipilimumab in lung and liver metastases of NSCLC, suggesting that liver SBRT led to greater T cell activity than lung SBRT, leading to better clinical benefit [73] (Fig. 2).

Moreover, hyperfractionated RT seems to be more effective than conventional fractionation because of its higher antitumor productive immunity [74]. Following the application of a mathematical model, conventional RT using 1.8–2 Gy fractions given five times a week over several weeks, primarily targeting the vulnerabilities of the tumor’s DNA damage response and cell cycle arrest mechanisms [47], was suggested to exert an immunosuppressive effect [61]. The favorable immunogenic modulation of a high dose fractionated daily RT dose compared with repeated exposure to conventional RT may also be explained by the IED, which simulates the intrinsic immunogenic activity of RT schedules [63]. In subcutaneous models of lung cancer and melanoma, ablative hypofractionated radiation therapy (23 Gy/2f) had greater efficacy than conventional fractionated radiation therapy (36 Gy/9f) in tumor growth inhibition and mouse survival improvement by reducing the accumulation of MDSCs into TME and decreasing their level of PD-L1 [75]. However, clinical studies have reported an antitumor immunomodulatory role for conventional fractionated RT, particularly when correlated to ICB [76]. Therefore, conventionally fractionated RT can not only contribute to an immunosuppressive TME by inducing TGF-β and IFN but also trigger an immune-supportive TME by inducing vasculature normalization [61] and the M1 macrophage phenotype [77]. Compared with conventionally fractionated RT, tumor microenvironment elements, including tumor hypoxia, T cell immune activity, vascular system, and inflammatory factors, all differ upon administration of hypo-fractionation RT [78]. Hence, understanding the distinct roles of conventional fractionation and hypo-fractionation on direct cancer cell killing and on the tumor microenvironment might have implications for the choice of combination therapies [79]. Overall, the RT scheme, i.e., the dose per fraction and consecutive fractions, can exert both immunostimulatory and immunosuppressive roles.

In addition to determining the optimal RT regimen, deciphering the optimal chronological sequence and the resting time between RT and ICBs is challenging. In a quantitative systems pharmacology model, prior or concurrent administration of PD-L1 blockade and RT elicited synergistic antitumor effects, which might result from more favorable dynamics between RT-triggered immune modulation and alleviated immune inhibition of T cells via PD-L1 blockade [80]. This study also indicates the vascular normalization role of ICB [36] and RT [81], which would mediate the crosstalk between normalization of tumor vasculature and stimulation of immune cell function, thus potentiating the efficiency of both RT and ICB. According to these data, ICB administration prior to or concurrent with RT might exert antitumor effects by overcoming immune resistance and potentiating antitumor immunity [61]. However, administration of anti-PD-1/PD-L1 agents should be most effective if administered after RT, while CTLA-4 should be administered prior to RT, as PD-L1 levels in tumor and immune cells increase following RT, and anti-CTLA-4 antibodies contribute to the depletion of intratumoral Tregs before RT to mitigate the immune suppression of TME [61]. For example, the KEYNOTE 001 trial reported that pembrolizumab treatment after RT among patients with advanced NSCLC led to a more favorable PFS and OS, relative to patients not administered pre-RT [82]. Similarly, in a retrospective study of patients with metastatic NSCLC, immunotherapy was administered at least 21 days after SBRT and presented better OS (19 months vs. 15 months, *P* = 0.0335) compared to immunotherapy within 21 days of SBRT [83]. In the PACIFIC trial investigating locally advanced NSCLC, durvalumab administration up to 6 weeks after chemoradiotherapy (chemotherapy plus concurrent radiation therapy) led to a prolonged median PFS of 11 months [76]. Therefore, it is critical to investigate why remote sequencing, instead of closer administration of immunotherapy, promotes tumor control within a certain context. Therefore, there remains no established consensus on the optimal timing and precise resting time for RT plus ICBs in the clinical setting. Optimal sequencing depends on the specific mechanism of T cell activation [29]. In addition, high-dose RT administered with double checkpoint inhibition should be more effective than targeting only one pathway [84]; however, it adds further complexity to sequencing and scheduling.

Notably, almost all preclinical and clinical data investigating RT plus immunotherapies have used external beam radiation therapy, while RT delivered using brachytherapy can achieve better dose conformality and dose heterogeneity. As it requires the insertion of a radioactive implants into the tumor tissue, brachytherapy may be an ideal method for achieving in situ tumor vaccination [85]. Therefore, brachytherapy provides an opportunity for locally delivering immunotherapy agents, in addition to the locally initiating RT [86].

Moreover, RT efficacy is reportedly associated with oxygen availability and tumor perfusion [10], providing an opportunity for combination therapy with anti-angiogenic agents. Anti-angiogenic therapies induce vasculature normalization, thus enhancing tumor perfusion and oxygenation [87], which in turn fosters the antitumor efficacy of RT. RT then promotes recruitment of effective T cells, such as cytotoxic CD8 and TH1 cells, into the TME via stimulation of chemokines, including CXCL9, CXCL10, and CXCL16, and via stimulation of cell-adhesion molecules ICAM-1 and VCAM-1. These vascular cell-adhesion molecules prompt the adhesion of lymphocytes to the vascular endothelium [61]. Notably, vascular normalization occurs only within a limited window, and the continuation of anti-angiogenic therapy ultimately leads to vasculature regression and decreased tumor oxygenation [2]. Furthermore, anti-angiogenesis can be beneficial when used prior to RT, as well as during or after RT [88], which seems to contradict the temporary character of vascular normalization.

Enhanced perfusion via anti-angiogenesis not only affects RT, but RT also influences perfusion due to its impact on the vasculature. High-dose RT was found to initially reduce tumor perfusion via the loss of endothelial cells and pericytes in a neuroblastoma xenograft model [89]. High-dose irradiation (≥10 Gy) elicits tumor endothelial cell death, leading to secondary killing of cancer cells via nutrient depletion, leading to deterioration of the tumor microenvironment [90]. For instance, subsequent tissue hypoxia can cause indirect tumor cell death [91], indicating that vascular disruption might not be an essential factor in tumor eradication. However, RT exerts dose-dependent effects on the tumor vasculature because of the angiogenic rebound effect. In a biomechanical model, doses of more than 10 Gy per fraction induced significant vascular collapse and reduced vascular flow and vascular radiation damage-related hypoxia [91]. High doses of RT (single dose of 14 Gy) can also trigger vasculogenesis via the influx of endothelial progenitor cells through recruitment of various chemokines (CXCL12/CXCR4) and by enhancing pericyte coverage on the endothelial tubes through the SDF-1α/CXCR4 and PDGF-B/PDGFR-β signaling [92]. Fractionated low-dose radiotherapy schedule, i.e., daily fractions of 2 Gy, seems to increase tumor vasculature formation and tissue perfusion in different tumor models and patients, due to reduced oxygen consumption, vasorelaxation via augmented inflammation, and enhanced growth of new blood vessels via pro-angiogenic factors such as VEGF and PlGF [10,93,]. Moreover, 10 Gy of irradiation was shown to stimulate the proliferation and migration of human umbilical vein endothelial cells, potentially promoting tumor vascularization [94]. Chemoradiation therapy (27 × 1.8 Gy) was also correlated with enhanced tumor blood volume in patients with cervical cancer [95]. For example, low-dose RT (28 × 1.8 Gy) can stimulate angiogenesis [96] and enhance vasculature normalization [97]. Collectively, the dose-scheduling of anti-angiogenic agents and RT impacts whether, and when, vasculature normalization or tumor perfusion is altered, as well as the angiogenic rebound effect occurs [10]. Therefore, it is critical to identify the optimal dose schedule for both therapeutic strategies to achieve the best clinical outcomes (Fig. 2B).

Hence, the positive role of a suitable RT dose on the vasculature and immune response provides a rationale for the triple combination of RT, ICBs, and anti-angiogenic therapy.

## Triple combination of ICBs, anti-angiogenic agents, and RT in a preclinical and clinical setting

As mentioned above, RT can induce vessel normalization and enhance the release and presentation of tumor antigens, drive infiltration of effector T cells into tumor tissue, and upregulate tumor PD-L1 and MHC-I expression. This upregulation can be overcome by the effects of ICB treatment. Anti-angiogenesis agents can promote trafficking of immune effector cells to the tumor sites and limit hypoxia partly via vessel re-normalization, drive DC maturation, reduce MDSCs and Tregs, and transiently augment perfusion, thereby radiosensitizing cancer cells and strengthening the efficiency of ICBs [10]. These dynamic interactions provide a rationale for the triple combination of ICB, RT, and anti-angiogenesis for cancer management. Here, we review the available data regarding triple combination therapy in preclinical settings among several cancer types (Tables 2 and 3).

**Table 2:** Preclinical studies testing triple combinations of anti-angiogenic therapy, immune checkpoint blockades, and radiotherapy among different solid cancer types.

Tumor model	Antiangiogenic therapy	ICB	RT	Immunological effects	Vasculature effects	Key results
Murine Lewis lung carcinoma cells	Anti-VEGF (100 µg on day 0, 3, 6, 9, total 400 µg)	Anti-PD-L1 (100 µg on day 1, 4, 7, 10, total 400 µg)	RT (40 Gy/4 fx on day 1, 2, 3, 4)	RT increased PD-L1 expression on CD8+ T, CD4+ T, dendritic, myeloid-derived suppressor cells, and tumor cells, increased PD-1 expression on CD8+ and CD4+ T cells; anti-angiogenic therapy insignificantly decreased the RT-induced PD-1 expression on CD8+ and CD4+ T cells; local accumulation of CD8+ T cells and reduction in MDSCs; increased the proportion of central memory T cells in splenocytes.	Transient vessel collapse was observed within 6 days after RT, and blood flow recovered at 1 week after RT.	Improved survival (*p* = 0.003)

*ICBs* immune checkpoint blockades, *RT* radiotherapy, *MDSCs* myeloid-derived suppressor cells.

**Table 3:** Clinical trials investigating combination of anti-angiogenic therapy, immune checkpoint blockades, and radiotherapy in patients with solid cancer.

Clinical trials gov number	Phase	Disease setting	Agents	RT	Primary endpoint	Estimated study completion date	Recruitment status	Patient population	most common AEs	AEs (total, Gr 3–5)
NCT04609293	Observational **s**tudy	Locally advanced/metastatic or recurrent RCCs	Camrelizumab+ Apatinib	Hyperfractionated RT (marginal dose of 50 Gy/2 Gy/25 f and tumor center dose of local hyperfraction 24–32 Gy/8–12 Gy/3–4 f)	ORR	2024	Not yet recruiting	30	–	–
NCT02313272	I	Recurrent HGGs	Pembrolizumab + bevacizumab	HFSRT (30 Gy/5 f)	Safety and tolerability	2020	Active, not recruiting	32	Proteinuria, increased alanine aminotransferase, fatigue, hypertension	Gr 3: 12 (34.4%)
NCT02829931	I	Recurrent HGGs	Ipilimumab+Nivolumab+ bevacizumab	HFSRT (30 Gy/5 f)	Safety and tolerability	2022	Recruiting	26	–	–

*RCCs* renal cell carcinomas, *HGGs* high-grade gliomas, *RT* radiotherapy, *HFSRT* hypofractionated stereotactic irradiation, *ORR* objective response rate, *AEs* adverse events, *Gr* grade.

### Renal cell carcinoma

A prospective, single-center, observational clinical trial (NCT04609293) was designed to explore the effectiveness and feasibility of combined camrelizumab, apatinib, and hyperfractionated RT in patients with locally advanced/metastatic or recurrent renal cell carcinoma (RCC), with ORR as the primary endpoint. All patients will be given camrelizumab in conjunction with apatinib until disease progression, intolerable toxicity, or a patient/investigator decision to stop treatment. Hypofractionated RT will be conducted one week after the second injection of camrelizumab. This study aimed to investigate whether SBRT can elicit an antitumor immune response and explore indicators that predict treatment efficacy in RCC, while the trial has not been conducted yet, with no results available.

### Lung cancer

Chen et al. reported that in a lung cancer mouse model, high-dose RT alone elicited radioresistance by upregulating PD-L1/PD-1 levels in tumor cells and in microenvironments [98]. Intriguingly, they further demonstrated that the addition of anti-VEGF treatment to high-dose ablative RT insignificantly overcame the immunosuppressive microenvironment, suggesting a weak reversal of the pro-tumor TME. In addition, adding anti-PD-L1 or anti-VEGF to high-dose RT generated memory immune response and vessel normalization, respectively, capable of preventing tumor recurrence and potentiating the RT antitumor response. Furthermore, the triple combination of anti-VEGF, RT, and anti-PD-L1 therapies more obviously enhances the existing anticancer efficacy. Overall, although their results did not reveal significant benefits when adding anti-VEGF to ablative RT in combination with anti-PD-L1 antibodies, the trimodal strategy indeed exhibited similar, yet more prominent, antitumor immune response than that of the dual modality (RT and anti-PD-L1).

### High-grade glioma

In a phase I trial, a triple combination of pembrolizumab, hypofractionated stereotactic irradiation (HFSRT), and bevacizumab was generally well tolerated, and a durable objective response was observed in half of the patients with recurrent high-grade glioma (HGG) [99]. ORRs of 83% (95% CI, 63–95) and 62.5% (95% CI, 24.5–91.5) occurred in bevacizumab-naive and bevacizumab-resistant patients, respectively. Disease control rates (complete response + partial response + stable disease) of 100% (95% CI, 85.8–100) and 75% (95% CI, 34.9–96.8) were observed among the bevacizumab naive and exposed groups, respectively [99]. Despite the small sample size and heterogeneous population, the antitumor activity of this combination modality is exciting.

Among bevacizumab-naive patients with HGG, an ongoing phase I study (NCT02829931) is investigating the combination of HFSRT (30 Gy in five fractions), bevacizumab, ipilimumab, and nivolumab, which might provide further information on the efficacy of ICB and anti-angiogenesis in combination with HFSRT.

### Other malignancies

Considering that VEGF levels are upregulated following RT treatment in HCC patients [100], it is rational to assess the benefits of anti-angiogenic and ICB therapy after RT because of their ability to enhance antitumor functions among HCC patients. One alternative approach might be that anti-angiogenic agents are administered before RT, thus normalizing the tumor vessel system, and in turn, fuel greater tumor-killing effects of RT [101]. This combinatorial approach of ICBs and anti-angiogenic agents might result in a greater window of vasculature normalization [23], which could be applied to enhance the effect of RT. While these results indicate potential for the aforementioned effects and require further validation in both preclinical and clinical studies, the best combinational agents, as well as the timing of RT and antibodies against PD1/PD-L1 and VEGF, are yet to be completely identified [102].

Overall, there are a small number of studies that have been performed in limited cancer types, and only Phase I clinical trials have been performed in a small number of cases, with a lack of validated prospective data. Therefore, further preclinical and clinical evidence from larger studies investigating the feasibility and efficacy of trimodal therapeutic strategies for other malignancies is warranted. Moreover, studies to enhance treatment efficiency by identifying ideal combination regimens, therapy sequencing, and RT dose/fractionation are needed.

## Biomarkers of combination of ICBs, anti-angiogenesis, and RT

It is pivotal to explore suitable predictive and prognostic markers, as well as immune and vasculature profiling techniques, for favorable patient selection and stratification. This would also contribute to identifying immunological and vascular correlations with treatment outcomes and to establishing personalized combination strategies to improve therapeutic efficacy.

Given the diversity and complexity of combination therapy, appropriate biomarkers are not likely to be adequate with just a single gene or protein [103], but they will require a multi-omics approach including genomic, transcriptomic, proteomic, metabolomic, and microbiomic investigations [104]. For example, readily accessible peripheral blood levels of circulating immune checkpoint proteins, cytokines, and antitumor autoantibodies may prove effective as biomarkers for predicting patient responses to RT and radioimmunotherapy [103]. Moreover, genetic profiling of ovarian cancer by next-generation sequencing (NGS) provides a better understanding of tumor heterogeneity and its potential role in determining the most appropriate treatment modality by identifying patients with distinct therapeutic vulnerabilities [105]. Tumor mutation burden, immune gene expression signatures, T-cell receptor repertoire, T-cell-inflamed gene expression, and microbiome by NGS can also be applied to predict radiosensitivity [106]. In addition, the index and gene signatures of radiosensitivity have been explored, but they fell short of predictive significance [107]. VEGF tyrosine kinase inhibitors also lack a robust biomarker for routine clinical usage [3]. As described above, the RT dose threshold for TREX1 induction at levels sufficient to degrade cytosolic DNA ranged from 12 to 18 Gy in several cancer types [62], suggesting that TREX1 might function as a biomarker to identify the most effective RT dose and fractionation.

Therefore, no biomarkers are currently validated for the prediction of a patient’s response to the triple combinational treatment, and all existing emerging biomarkers require further examination in preclinical studies and validation in clinical trials.

## Safety of combined ICBs, anti-angiogenesis, and RT

Regarding safety assessments, in mice with lung cancer, trimodal therapies of anti-VEGF, anti-PD-L1, and RT were generally tolerated without serious toxicity [98]. In addition, a small phase I trial combining pembrolizumab, HFSRT, and bevacizumab reported that the most common adverse events (AEs) for grade 3 occurred in 12 (34.4%) HGG patients, with hypertension and thromboembolism being the most common. The reason for therapy discontinuation in only one patient was asymptomatic grade 3 elevated aspartate aminotransferase levels. Systemic corticosteroids were applied in only two patients because of immune-related AEs. Therefore, it is not feasible to evaluate the effect of corticosteroid use on treatment response, owing to the small number of patients [99] (Table 3).

The balance between benefits and risks requires further exploration. For example, high doses of RT can cause damage to adjacent normal tissues, generally leading to severe AEs, such as serious radioactive gastritis, esophagitis, pneumonitis, liver function abnormalities, and intracranial radiation necrosis [66]. However, this effect can be partially alleviated by hypofractionated RT. For example, gastrointestinal toxicities markedly decreased with a multi-fraction (25–45 Gy in 3–5 fractions) compared to a single-fraction regimen (25 Gy in one fraction) for SBRT in pancreatic tumors [66]. Among pancreatic tumors, the rates of lymphopenia following SBRT and conventional fractionation RT were 13.8% and 71.7%, respectively [108].

Numerous anti-angiogenesis agent-associated toxicities have been reported, primarily including cardiovascular and non-cardiovascular adverse effects, depending on the category of prescribed drug [11]. Considering the increased AEs when adding immunotherapy to RT [109], as well as the enhanced RT-related gastrointestinal luminal toxicities when adding angiogenic inhibitors to RT [110], it is conceivable that even more pronounced AEs may be noted by adding RT to the double combination of anti-angiogenesis with ICBs.

The propensity to develop immune-related AEs after combinational therapeutic strategies may be affected by the specific site and nature of the tumor and may be more obvious when overlapping AEs are noted. For example, the additive effect of lung injury was observed when ICBs were combined with RT in patients with lung cancer [111]. Similarly, different cancer types, choice of ICB, tumor histology, and mutational burden are associated with distinct AEs [74]. Moreover, the heterogeneity of anti-angiogenic receptor tyrosine kinase inhibitors may correspond to different pharmacokinetics and substance-specific AEs, potentially resulting from the variable affinity and potency to targets due to different chemical structures [112].

Given that the triple combination field is in its early stages, only two studies have shown tolerable toxicity profiles in lung cancer and HGGs, respectively, while the combination did not specify the best RT dose, fractionation scheme, or RT/ICBs/anti-angiogenesis sequence. Therefore, it is too early to determine whether this strategy is feasible. However, it has become increasingly important to explore biomarkers for treatment responses, thus optimizing treatment efficacy and minimizing treatment toxicity.

## Conclusion and future direction

RT, angiogenesis inhibitors, and ICBs all influence both the tumor vasculature and tumor-killing immunity. Based on the available preclinical and clinical data regarding the addition of RT to the blockades of angiogenesis or immune checkpoints, the triple combination appears to offer an effective cancer treatment model for the future. Similarly, other immunotherapy strategies, including immunostimulatory factors, autologous immature DCs, vaccination, or TLR agonists in conjunction with RT, have shown promising results among various tumor types [10]. Among these therapeutic strategies, intratumoral injection of TLR agonists or autologous immature DCs plus RT presented excellent safety and tolerability [10]. Therefore, RT plus immunotherapy seems promising, but adding anti-angiogenic therapy to the dual combination presents additional complexity regarding dose-scheduling, timing, and potential toxicities. In addition to anti-angiogenic therapy, RT and ICBs after surgery have shown promising antitumor activity [113]. Until now, the combination of RT, immunotherapy, and surgery in either the adjuvant or neoadjuvant setting has been largely ignored. Further preclinical research should stress the great clinical importance of the combination and clarify its distinct immunological features, such as a highly reduced tumor antigen load in the adjuvant setting [86].

Therefore, we are a long way from having triple combination therapy adopted as a frontline treatment in clinical practice. First, the timing, dose, duration, treatment sequences, and reagent selection for the therapies must be determined, as these may directly affect systemic antitumor immunity. Moreover, based on the limited data available regarding the tolerable synergistic cytotoxicity, the triple combination appears promising; however, safety remains largely unexplored and requires further analysis. It is also challenging to choose a suitable patient population because some cancer types have been shown to be relatively resistant to certain therapeutic approaches. In addition, the differences in vascular normalization mediated by anti-angiogenic therapy, ICBs, and RT require further clarification.

Overall, with the limited clinical trials and even fewer preclinical data, there remains a large gap in knowledge regarding how best to utilize these strategies to optimize patient benefits, while limiting potential adverse effects. With the complexity of the trimodal combination, the only way to effectively identify the optimal combination is through an enhanced understanding of how each individual treatment alters the tumor microenvironment, as well as how to best balance immunosuppressive and immune permissive environments. Further investigation regarding predictive and prognostic biomarkers is also needed to allow for optimal patient population recruitment.
